# Human Epicardial Adipose Tissue cTGF Expression is an Independent Risk Factor for Atrial Fibrillation and Highly Associated with Atrial Fibrosis

**DOI:** 10.1038/s41598-018-21911-y

**Published:** 2018-02-26

**Authors:** Qing Wang, Wang Xi, Liang Yin, Jing Wang, Hua Shen, Yang Gao, Jie Min, Yufeng Zhang, Zhinong Wang

**Affiliations:** Center for Comprehensive Treatment of Atrial Fibrillation, Department of Cardiothoracic Surgery, Changzheng Hospital, Second Military Medical University, Shanghai, China

## Abstract

Epicardial adipose tissue (EAT) is associated with the incidence, perpetuation, and recurrence of atrial fibrillation (AF), with elusive underlying mechanisms. We analyzed adipokine expression in samples from 20 patients with sinus rhythm (SR) and 16 with AF. Quantitative real-time PCR showed that connective tissue growth factor (cTGF) expression was significantly higher in EAT than in subcutaneous adipose tissue (SAT) or paracardial adipose tissue (PAT) from patients with AF, and in EAT from patients with SR (P < 0.001). Galectin-3 expression was significantly higher in EAT than in SAT or PAT (P < 0.001), with no significant differences between patients with AF and SR (P > 0.05). Leptin and vaspin expression were lower in EAT than in PAT (P < 0.001). Trichrome staining showed that the fibrosis was much more severe in patients with AF than SR (P < 0.001). We found a linear relationship between cTGF mRNA expression level and collagen volume fraction (y = 1.471x + 27.330, P < 0.001), and logistic regression showed that cTGF level was an independent risk factor for AF (OR 2.369, P = 0.027). In conclusion, highly expressed in EAT, cTGF is associated with atrial fibrosis, and can be an important risk factor for AF.

## Introduction

Atrial fibrillation (AF), the most common sustained arrhythmia in the clinic, has raised worldwide concern due to its increasing incidence, morbidity and mortality^1^. Recent clinical studies have found that epicardial adipose tissue (EAT) is highly associated with the incidence^2^, perpetuation^3^ and recurrence^4^ of AF. The predominant views consider paracrine effects through the release of adipokines, the infiltration of adipocytes into the atrial myocardium, and the source of myofibroblasts producing extracellular matrix as the major mechanisms by which EAT facilitates AF^5^. However, the precise role that EAT plays in the pathogenesis of AF is not yet been completely understood, and therefore requires further study.

Structural remodeling is a key process in the development and perpetuation of AF, which is manifested by fibrosis^6^. A study demonstrated that EAT could induce atrial fibrosis via secretion of Activin A, which contributed to the development of AF^7^. However, whether other adipokines are of importance in the relationship between EAT and AF is still unknown. It was supposed in this study that some adipokines expressed in EAT could facilitate the process of atrial remodeling. We screened previous reports to find potential adipokines that could be expressed in adipose tissue and associated with fibrosis. Connective tissue growth factor (cTGF)^8,9^, galectin-3 (gal-3)^10,11^, leptin^12,13^ and vaspin^14,15^ were chosen as the candidates of this hypothesis. Previous research studied the link between mRNA level of chemerin^16^ or omentin-1^17^ in EAT and coronary atherosclerosis, which also demonstrated the feasibility and reliability of mRNA quantification. Hence, quantitative real-time PCR was firstly used to determine the mRNA expression level of the adipokines, while immunochemistry and western blotting were further adopted to validate the results. This study compared the expression of cTGF, gal-3, leptin and vaspin in three types of adipose tissue between AF patients and sinus rhythm (SR) patients undergoing coronary artery bypass graft (CABG) surgery. In addition, the quantitative relationship between adipokine expressions, atrial fibrosis and AF was analyzed.

## Results

### Patient characteristics

There was no significant differences between SR patients and AF patients in demographical data including age (P = 0.741), sex (P = 0.821), Body Mass Index (BMI) (P = 0.631), or smoking status (P = 0.936). Patients showed no disparity in cardiac function, as indicated by NYHA functional class or left ventricular ejection fraction (LVEF). However, echocardiography showed that the left atrial diameter (LAD) was significantly higher in the AF group than in the SR (P = 0.004). In terms of comorbidity, there was no significant difference between the two groups, including hypertension (P = 0.439), type 2 diabetes mellitus (T2DM) (P = 0.517), stroke (P = 0.418) and chronic obstructive pulmonary disease (COPD) (P = 0.451; see Table 1 for details).

**Table 1:** Clinical characteristic of patients in the AF and SR group.

Variables	SR group (n = 20)	AF group (n = 16)	χ^2^/t	P
Demographics				
Age(y)	58.6 ± 9.4	57.5 ± 9.1	0.333	0.741
Gender(%male)	12 (60.0%)	9 (56.3%)	0.051	0.821
BMI(kg/m^−2^)	23.4 ± 2.1	23.7 ± 2.4	0.485	0.631
Smoking	6 (30.0%)	5 (31.3%)	0.007	0.936
NYHA functional class			1.190	0.979
I	1 (5.0%)	1 (6.3%)		
II	10 (50.0%)	8 (50.0%)		
III	7 (35.0%)	6 (37.5%)		
IV	2 (10.0%)	1 (6.3%)		
Echocardiography				
LVEF(%)	58.2 ± 3.9	57.7 ± 4.8	0.319	0.751
LAD (mm)	38.5 ± 5.4	43.3 ± 3.6	3.078	0.004
Comorbidities				
Hypertension	6 (30.0%)	3 (18.8%)	0.600	0.439
Type II Diabetes	7 (35.0%)	4 (25.0%)	0.419	0.517
Stroke	1 (5.0%)	2 (12.5%)	0.655	0.418
COPD	2 (10.0%)	3 (15.0%)	0.569	0.451

Data are presented as n (%) or mean ± SD. BMI, body mass index; LVEF, left ventricular ejection fraction; LAD, left atrial diameter; T2DM, type 2 diabetes mellitus; COPD, chronic obstructive pulmonary disease; NYHA, New York Heart Association.

### Adipokine mRNA expression

**Figure 1:**
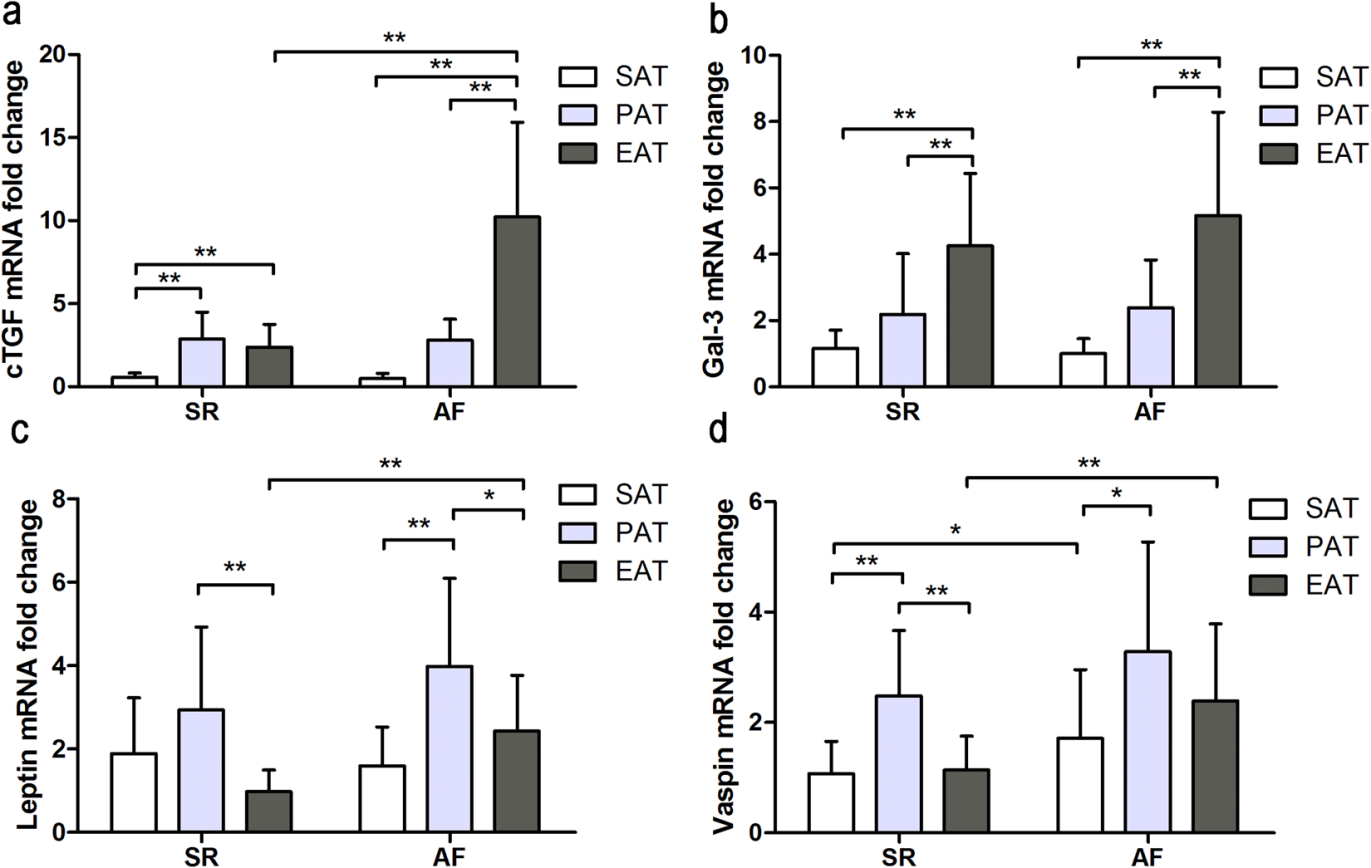
Quantitative real-time PCR results of adipokines expression in the adipose tissue of patients with AF (n = 16) and SR (n = 20). (**a**) Quantitative real-time PCR results of cTGF; (**b**) Quantitative real-time PCR results of gal-3; (**c**) Quantitative real-time PCR results of leptin; (**d**) Quantitative real-time PCR results of vaspin; *P = 0.001, **P < 0.001.

Adipokine mRNA expression is shown in Fig. 1 and Supplemental Table S1. cTGF mRNA expression was much higher in EAT from AF patients, compared to subcutaneous adipose tissue (SAT) and paracardial adipose tissue (PAT), as well as EAT from SR patients (AF EAT vs. AF SAT, 10.23 ± 5.69 vs. 0.49 ± 0.32, P < 0.001; AF EAT vs. AF PAT, 10.23 ± 5.69 vs. 2.80 ± 1.28, P < 0.001; AF EAT vs. SR EAT, 10.23 ± 5.69 vs. 2.39 ± 1.38, P < 0.001).

Both groups showed comparable trends in gal-3 mRNA expression, which was manifested in sequential increments in SAT, PAT and EAT (SR F = 17.741, P < 0.001; AF F = 17.831, P < 0.001). However, there was no significant difference in gal-3 mRNA expression between the two groups (SAT, P = 0.306; PAT, P = 0.722; EAT, P = 0.314).

In terms of leptin, the highest expression was observed in PAT from both groups, which was significantly higher than that in SAT or EAT (AF PAT vs. SAT, 3.98 ± 2.12 vs. 1.60 ± 0.93, P < 0.001; AF PAT vs. EAT, 3.98 ± 2.12 vs. 2.43 ± 1.34, P = 0.020; SR PAT vs. SAT, 2.93 ± 1.99 vs. 1.88 ± 1.34, P = 0.068; SR PAT vs. EAT, 2.93 ± 1.99 vs. 0.98 ± 0.52, P < 0.001). Leptin expression in EAT was higher in the AF group than that in the SR group (P < 0.001).

Vaspin expression demonstrated a trend similar to that of leptin expression, with the highest mRNA expression levels in PAT (SR F = 17.820, P < 0.001; AF F = 3.997, P = 0.025), and vaspin expression in SAT and EAT was higher in the AF group than that in the SR group (AF SAT vs. SR SAT, 1.71 ± 1.25 vs. 1.07 ± 0.59, P = 0.049; AF EAT vs. SR EAT, 2.39 ± 1.39 vs. 1.14 ± 0.61, P = 0.001).

### Adipokine protein expression

**Figure 2:**
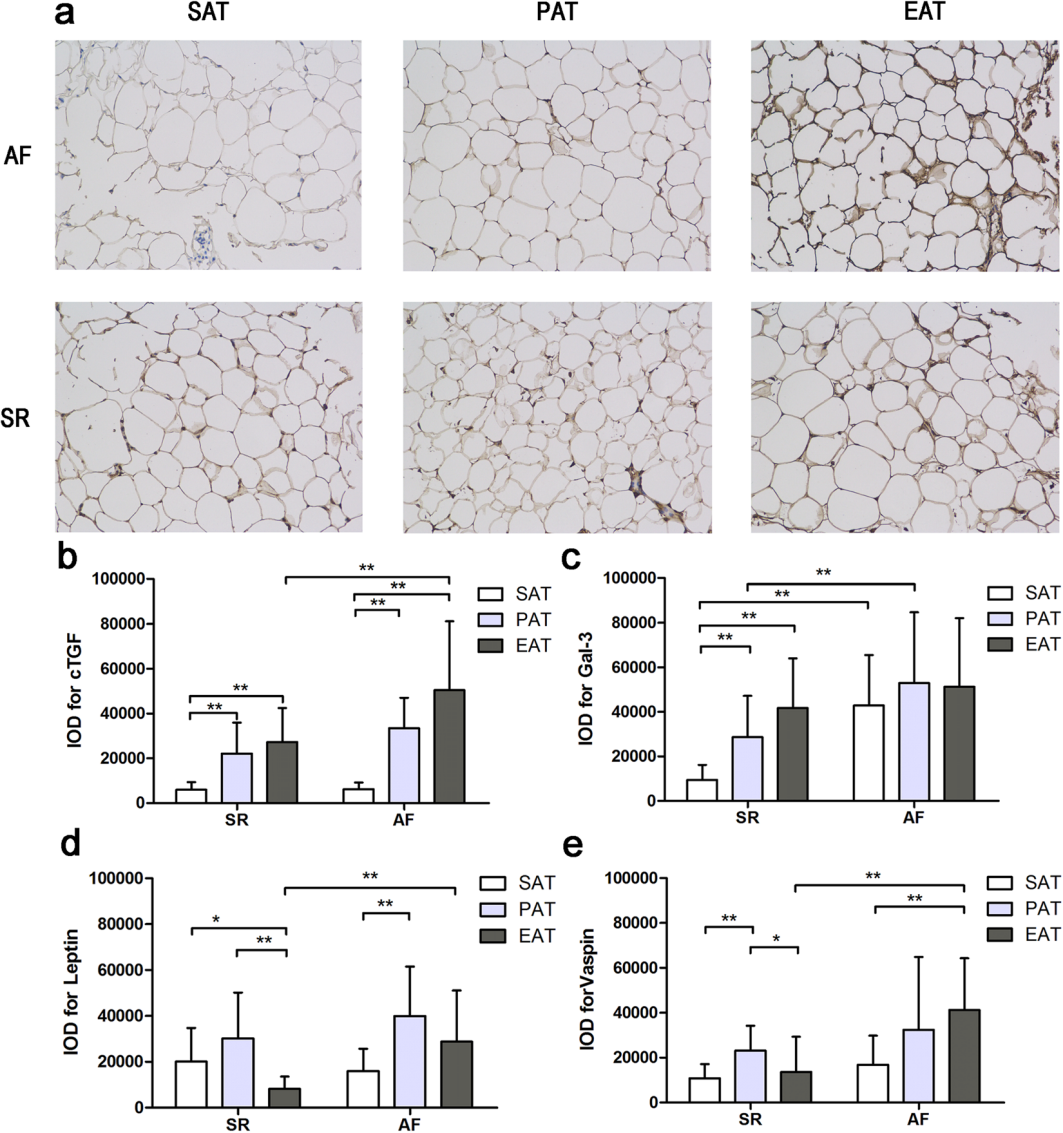
Immunohistochemical integrated optical density analysis of adipokines in the adipose tissue of patients with AF (n = 16) and SR (n = 20). (**a**) Representative sections cTGF expression of SAT, PAT, and EAT in the AF and SR groups (×200); (**b**) Quantitative results of IOD in sections of cTGF in the AF and SR groups; (**c**) Quantitative results of IOD in sections of gal-3 in the AF and SR groups; (**d**) Quantitative results of IOD in sections of leptin in the AF and SR groups; (**e**) Quantitative results of IOD in sections of vaspin in the AF and SR groups. *P < 0.05, **P < 0.01.

**Figure 3:**
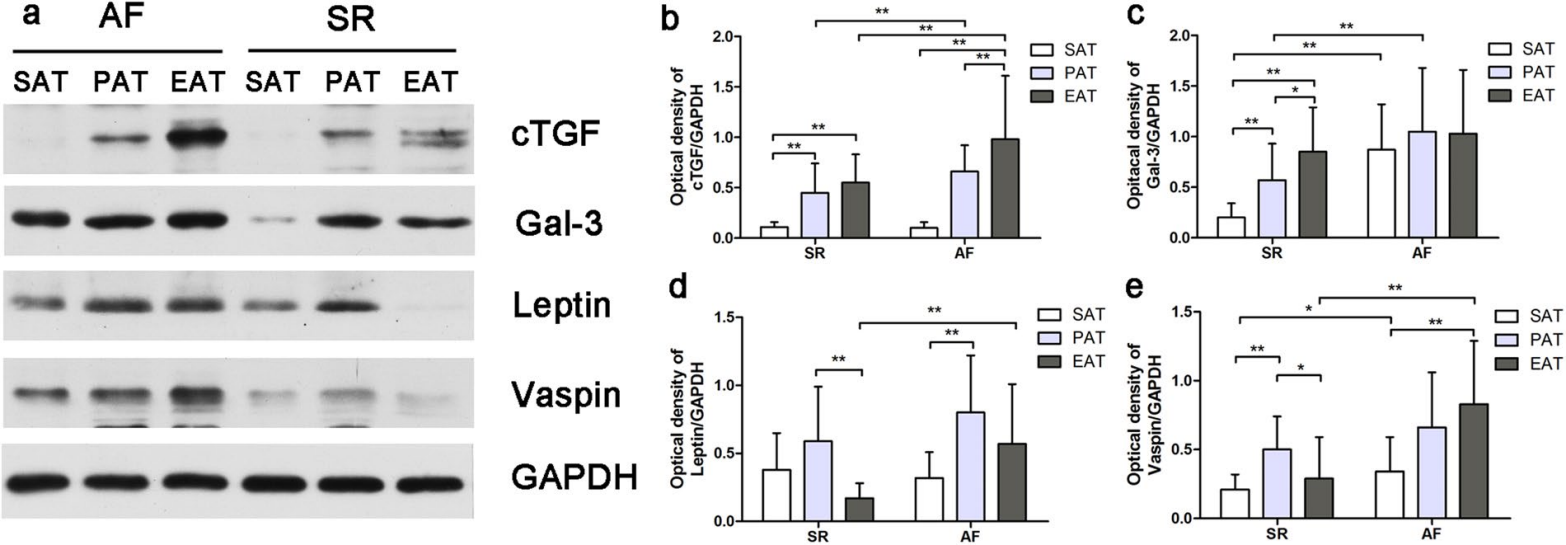
Western blotting and quantitative results of optical density of adipokines in the adipose tissue of patients with AF (n = 16) and SR (n = 20). (**a**) Western blotting of representative bands from SR and AF groups (full-length gels and blots are included in the Supplementary Information Fig. 1); (**b**) Quantitative results of optical density of cTGF; (**c**) Quantitative results of optical density of gal-3; (**d**) Quantitative results of optical density of leptin; (**e**) Quantitative results of optical density of vaspin. *P < 0.05, **P < 0.01.

As shown in Fig. 2 and Supplemental Table S2, adipokine protein expression was assayed qualitatively via immunohistochemistry and quantitatively using IOD values. Figure 2a shows representative slides of cTGF staining, which correlate with cTGF mRNA expression. EAT from patients with AF showed much more intense staining than SAT or PAT from the same patient. There were only relatively mild differences between SAT, PAT and EAT from the SR group. As depicted in Fig. 2b, quantitative IOD also confirmed that cTGF expression was significantly higher in EAT from the AF group (AF EAT vs. AF SAT, 50487.00 ± 30750.84 vs. 6240.7 5 ± 3001.37, P < 0.001; AF EAT vs. AF PAT, 50487.00 ± 30750.84 vs. 33461.50 ± 13565.64, P = 0.052; AF EAT vs. SR EAT, 50487.00 ± 30750.84 vs. 27277.85 ± 15141.24, P = 0.006). Western blotting results are shown in Fig. 3 and Supplemental Table S3. Results were consistent with the immunohistochemistry data, where cTGF was highly expressed in EAT from patients with AF (AF EAT vs. AF SAT, 0.98 ± 0.63 vs. 0.10 ± 0.06, P < 0.001; AF EAT vs. AF PAT, 0.98 ± 0.63 vs. 0.66 ± 0.26, P = 0.088; AF EAT vs. SR EAT, 0.98 ± 0.63 vs. 0.55 ± 0.28, P = 0.011).

As for gal-3 expression, the immunohistochemistry and western blotting results were slightly different from the mRNA expression. There was no difference in gal-3 expression between SAT, PAT, and EAT from patients with AF (F = 0.564, P = 0.573) (Figs 2c and 3c). For leptin expression, protein levels showed a similar distribution to mRNA expression in adipose tissues (Figs 2d and 3d). Vaspin expression was higher in EAT from patients with AF than those with SR, which differs from the quantitative real-time PCR results (Figs 2e and 3e).

### Correlation between atrial fibrosis levels and adipokine expression

**Figure 4:**
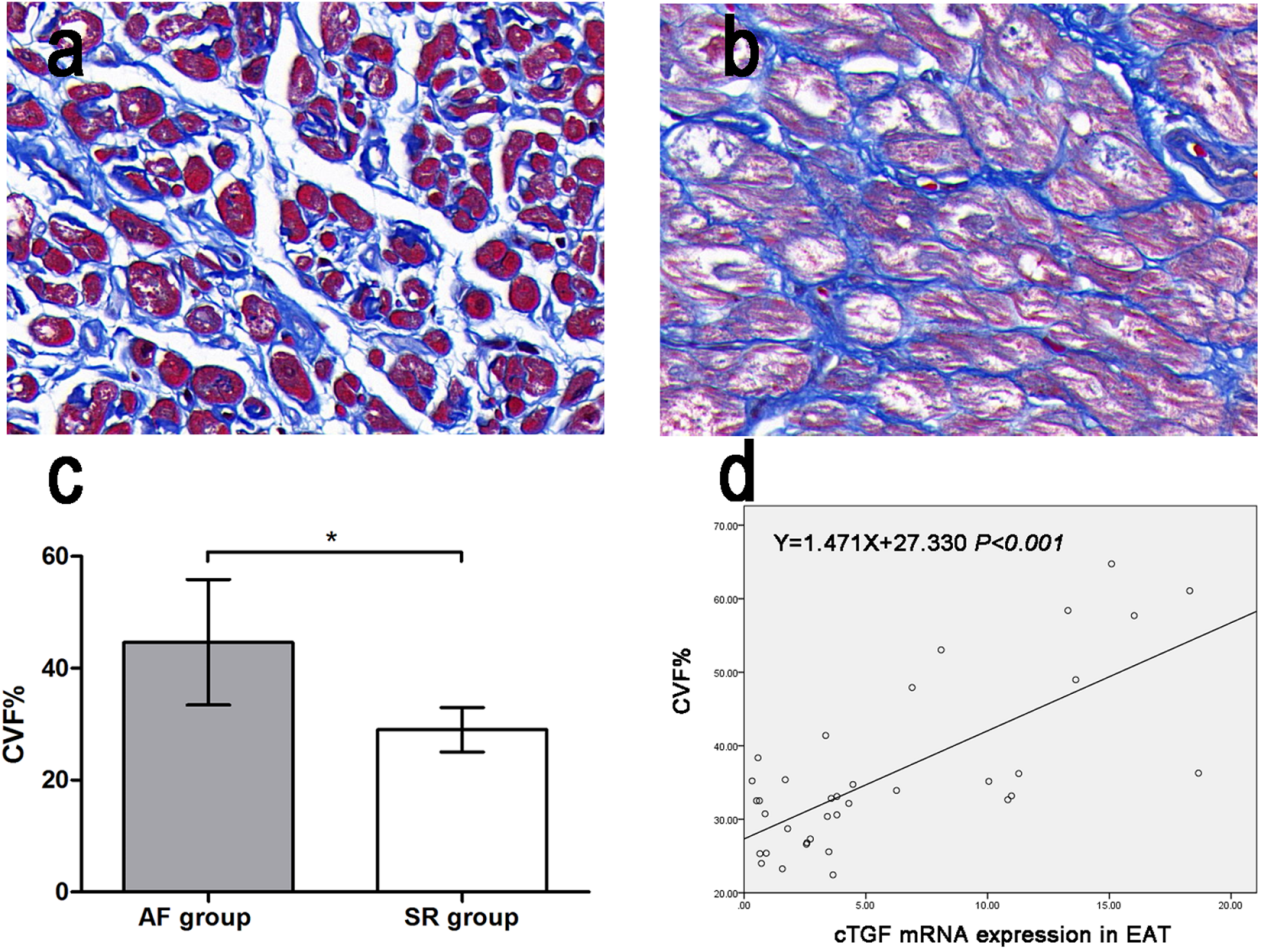
Masson staining and quantitative results of atrial myocardium from patients with AF (n = 16) and SR (n = 20). (**a**) Masson staining of representative sections from the SR group(×400). (**b**) Masson staining of representative sections from the AF group(×400). (**c**) Quantitative results of Masson staining. *P < 0.001. (**d**) Univariate linear regression analysis of cTGF mRNA expression and the collagen volume fraction (CVF%).

Masson’s-stained sections of the atrial myocardium are shown in Fig. 4a,b. Representative sections indicate a more fibrotic and disordered myocardium in the AF group than in the SR group. As shown in Fig. 4c, the quantitative CVF% in the AF group was significantly higher than that in the SR group (44.64 ± 11.23 vs. 29.02 ± 3.98, t = 5.798, P < 0.001). Univariate linear regression analysis of CVF% and adipokines revealed a linear relationship between cTGF mRNA expression and CVF% (y = 1.471 × + 27.330, P < 0.001), as shown in Fig. 4d. There was no significant linear relationship between CVF% and other adipokines analyzed, including gal-3, leptin and vaspin (Table 2).

**Table 2:** Curve estimation parameters of adipokines expression and CVF%.

Adipose tissue type	adipokines	Constant	b1	R^2^	F	P
SAT	cTGF	38.718	−5.134	0.017	0.577	0.453
	gal-3	40.666	−4.321	0.039	1.367	0.250
	vaspin	33.903	1.517	0.018	0.610	0.440
	leptin	38.055	−1.190	0.016	0.539	0.468
PAT	cTGF	36.754	−0.278	0.001	0.045	0.834
	gal-3	37.728	−0.776	0.013	0.454	0.505
	vaspin	30.568	1.901	0.076	2.797	0.104
	leptin	33.155	0.825	0.024	0.826	0.370
EAT	cTGF	27.330	1.471	0.530	38.328	<0.001
	gal-3	35.458	0.108	0.001	0.022	0.882
	vaspin	33.214	1.619	0.030	1.059	0.311
	leptin	33.341	1.614	0.030	1.063	0.310

### Multivariate regression analysis

We conducted a multivariate logistic regression analysis, when AF was defined as the dependent variable, while adipokine expression level and demographic data were included as candidates of independent variables. Enter method was used to choose independent varibles for the best model. Results showed cTGF mRNA expression was left in the model, and it was an independent risk factor for the incidence of AF (OR 2.369, 95%CI (1.104–5.087), P = 0.027), as shown in Table 3. A linear regression analysis was also conducted to explore the clinical parameters affecting the secretory profile, which showed that only AF was statistically associated with the mRNA expression of cTGF, leptin and vaspin (Supplemental Table S4).

**Table 3:** Multivariate logistic regression of risk factors for incidence of AF.

Risk factors	OR(95%CI)	P
Gender male	1.415 (0.074–23.414)	0.852
Age	1.056 (0.892–1.249)	0.528
Smoking	6.008 (0.095–380.069)	0.397
BMI	1.008 (0.561–1.812)	0.978
NYHA	0.174 (0.007–4.178)	0.281
T2DM	0.562 (0.026–12.299)	0.715
Hypertension	0.116 (0.002–5.955)	0.284
COPD	0.776 (0.006–100.081)	0.919
Stroke	3.192 (0.034–298.314)	0.616
cTGF mRNA in EAT	2.369 (1.104–5.087)	0.027

BMI, body mass index; NYHA, New York Heart Association; T2DM, type 2 diabetes mellitus; COPD, chronic obstructive pulmonary disease.

## Discussion

A strong relationship between atrial fibrosis and AF has been reported, and atrial fibrosis is believed to be a key process in structural remodeling of the atrium^18^. As previously shown, cardiac fibroblasts are activated by cytokines secreted by inflammatory cells, including macrophages, through the TGF-β signalling pathway, causing them to produce collagen I and collagen III, and ultimately leading to atrial fibrosis^19^. EAT, once thought to have a mere protective and metabolic role in the cardiovascular system, has recently been found to be highly associated with cardiovascular diseases, including coronary artery disease, heart failure^20^, and AF^21^. Previous work has suggested that EAT plays a key role in atrial remodeling by infiltrating into the myocardium^22^ and secreting Activin A, thus predisposing the tissue to atrial fibrosis^7^. We hypothesized that there were still other adipokines highly expressed and secreted by EAT and associated with atrial fibrosis, which might participate in the development of AF. Our study mainly focused on four potential adipokines, including cTGF, gal-3, leptin and vaspin, and the results showed that cTGF expression level was higher in EAT than other types of adipose tissue, and in EAT of AF patients than SR patients, which correlated well with our hypothesis.

**Figure 5:**
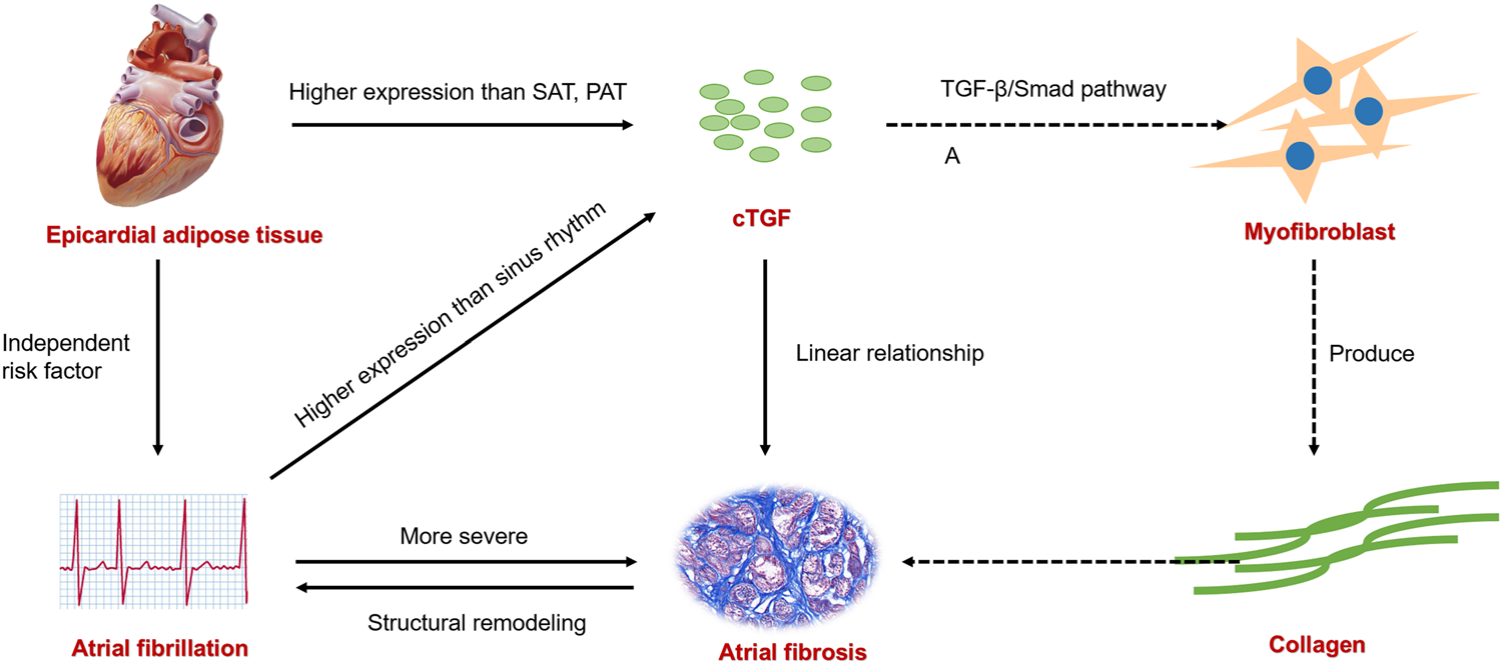
Visual illustration of the major findings and hypothesis.

As an versatile cytokines, cTGF is involved in a range of biological processes, including cell growth and differentiation^23^. A clinical study showed that serum cTGF level was an important predictor of recurrence of AF after the catheter ablation, implying the role of cTGF in atrial remodeling^24^. An experimental study using rapid pacing canine models also found that cTGF was up-regulated in AF models, providing further evidence that cTGF was associated with AF^25^. Not only working in circulation system, it is also highly expressed in subcutaneous or epididymal fat pads in an obesity mouse model^8^. Our study demonstrated that cTGF was highly expressed in EAT, whether with or without AF, suggesting that cTGF might be an important adipokines participating in the regulation of cardiac remodeling. We compared the cTGF expression level in EAT between AF patients and SR patients, and analyzed the risk factors for AF. The results strongly suggested the association between cTGF and AF. Supporting our hypothesis, a strong connection between cTGF and atrial fibrosis was established by linear regression analysis. Other studies also favors our study. One found that cTGF mainly acted in angiotensin II-mediated atrial fibrosis^26^, while other studies proved that it was regulated by miR-132^27^ and functioned via TGF-β1/Smad pathway^28^. Therefore, it is inferred that EAT is an important source of cTGF, which facilitates atrial fibrosis and atrial remodeling and further leads to AF (Illustrated by Fig. 5).

About other adipokines investigated in this study, gal-3 showed no difference between AF patients and SR patients, while leptin and vaspin were more expressed in PAT than EAT, which were not in favor of the hypothesis. Gal-3 is a member of the galectin family and is expressed extensively in macrophages, mastocytes and neutrophils^29^. In recent years, gal-3 has emerged as a promising biomarker associated with atrial fibrosis and AF^30^. However, its expression in EAT has yet been described. Despite higher gal-3 expression in EAT than SAT and PAT, there was no disparity between AF patients and SR patients. Immunohistochemistry and western blotting yielded slightly different results than for mRNA expression, which could be attributed to differences between mRNA and protein expression or sample error. We attributed the similarity between SR and AF patients in gal-3 expression level to the following potential reasons. On one hand, gal-3 might functioned in atrial fibrosis majorly via circulation system rather than paracrine pathway, which was validated by the higher serum gal-3 level in AF than SR patients. On the other hand, gal-3 expressed in EAT could be a part of inflammation of adipose tissue itself rather than secretory products of EAT^31^. Leptin was considered one of the most promising candidates for having a role in AF, because previous work verified its strong relationship with AF^13^ and because of its high expression in EAT^32^. The present study showed that leptin expression was higher in EAT from patients with AF than those with SR, which correlated with previous studies. However, we found that leptin expression was lower in EAT than PAT, whether for patients with AF or SR. A similar conclusion can be drawn from the vaspin expression results, another adipokine associated with cardiovascular disease^15^. The higher expression of vaspin and leptin in PAT might be attributed to the association of obesity. A study found that vaspin and leptin level were associated with adipose tissue depot and the insulin resistance, especially for visceral adipose tissue^33^. Notably, the significant difference in leptin and vaspin expression in EAT between patients with AF and SR suggested that they might still be involved in atrial fibrosis via EAT in a paracrine manner. A recent study showed a higher degree of local tissue hypoxia and up-regulation of leptin expression in the perivascular adipose tissue belonging to EAT, along with increased vascularization, inflammation, and fibrosis, which might contribute to the increased atherosclerotic plaque burden in the coronary arteries^34^. Similar procedures may happen in peri-atrial adipose tissue, where leptin expression is increased, inflammation and fibrosis was induced, which contributed to atrial remodeling and AF.

Our findings not only add more evidence of the negative role that EAT plays in the development of AF, but demonstrate that cTGF is highly expressed in EAT of AF patients and may facilitate atrial fibrosis, which help elucidate the underlying mechanism of AF and provide more clues for prevention work as well as therapy research. Although a lot of work has been carried out, and some intriguing findings have been acquired, some limitations with the current study should also be noted. Our work revealed differences in expression between SAT, PAT, and EAT from patients with AF and SR; however, further clinical and basic studies will be required to determine the exact role of these adipokines in the pathogenesis of AF. Second, multiple factors cannot be completely eliminated, although most major clinical characteristics were uniform across all groups. Lastly, this study only focused on the four most prominent adipokines, and a more extensive screen will be necessary in order to explore additional potential adipokines.

In conclusion, our study demonstrates that cTGF is highly expressed in EAT from patients with AF and is associated with atrial fibrosis. cTGF expression in EAT may also represent an independent risk factor for AF. Future studies aimed at understanding the role of cTGF in structural and electrical remodeling of the atrium could identify novel targets for the prevention and treatment of AF.

## Methods

### Human subjects

From March 2016 to April 2017, a total of 68 patients received CABG surgery in Changzheng Hospital affiliated with the Second Military Medical University. Of these, 36 patients were enrolled in this study. Patients were divided into SR group (n = 20) and AF group (n = 16), according to their heart rhythm. All 36 patients were enrolled according to the following inclusion and exclusion criteria: patients aged between 18 to 80 years receiving CABG with SR or AF rhythm were included, while patients with structural heart disease, paroxysmal AF rhythm, severe hepatic or renal dysfunction, metabolic disease, infectious disease, cancer, or aged over 80 years were excluded. Persistent and permanent AF was determined using a 12-lead electrocardiogram. Clinical data from all patients were collected for analysis.

This study was approved by the Committee on Ethics of Biomedicine of the Second Military Medical University. This study also complied with the Declaration of Helsinki, and signed, written informed consent was obtained from all subjects.

### Sample acquisition

All patients received a conventional on-pump CABG with a sternal incision. Adipose tissue was acquired during surgery, before cardiopulmonary bypass establishment. Different types of adipose tissue (average 0.5 g each) were collected from each patient, including SAT from the chest incision site, PAT from the pericardium, and EAT from the atrioventricular groove next to the right atrial appendage. Each biopsy was divided into 2 portions; one was frozen immediately at −80 °C for RNA isolation, and the other was immersed in neutralized formalin for immunohistochemistry. Adjacent atrial myocardial tissue (average 0.1 g) from the right atrial appendage tissue was also collected and likewise divided into 2 portions.

### RNA isolation and quantitative real-time PCR

TRIzol® reagent (Invitrogen, Carlsbad, CA, USA) was used to extract total RNA. Purity of the isolated RNA was assessed by measuring optical density at 260 nm and 280 nm. Reverse transcription was performed using a High-Capacity cDNA Reverse Transcription Kit (Applied Biosystems, Foster City, CA, USA), according to the manufacturer’s instructions. SYBR® Premix Ex Taq™ (Takara Bio, Tokyo, Japan) was used to prepare samples for quantitative real-time PCR. An ABI Prism 7900 Detector System (Applied Biosystems) was used to perform quantitative real-time PCR. Primers were designed using Primer Premier 6.0 software (Premier Biosoft, Palo Alto, CA, USA). Primer sequences were as follows: cTGF (cTGF), forward 5′-ATGCTGCGAGGAGTGGGTGT-3′, reverse 5′-TGGCTCTAATCATAGTTGGGTCT-3′; Gal-3 (LGALS3), forward 5′-GTGAAGCCCAATGCAAACAGA-3′, reverse 5′-AGCGTGGGTTAAAGTGGAAGG-3′; Leptin (LEP), forward 5′-CCTGTGCGGATTCTTGTGG-3′, reverse 5′-GGTGACTTTCTGTTTGGAGGA-3′; Vaspin (SERPINA12), forward 5′-CGGTGAAAGGTCTTCTAAAGCC-3′, reverse 5′- AGCCTAAGTCCATGTTCTGCC-3′; β-actin (ACTB), forward 5′-CATGTACGTTGCTATCCAGGC-3′, reverse 5′-CTCCTTAATGTCACGCACGAT-3′. Relative gene expression was calculated using threshold cycle value (C_T_) and formula 2^−ΔΔCT^.

### Immunohistochemistry

Paraffin-embedded sections were placed at 60 °C for 1 h, and then dewaxed, rehydrated and rinsed two to three times with PBS, for 3 min each. Sections were incubated in a 3% hydrogen peroxide solution for 8 min at room temperature, then rinsed 3 times with PBS (pH 7.2 to 7.6), for 5 min each. Sections were incubated with primary antibodies (anti-cTGF, anti-Gal-3, or anti-LEP, anti-Vaspin; Abcam, Cambridge, UK) overnight at 4 °C in a moist chamber, then rinsed 3 times with PBS (pH 7.2 to 7.6). Sections were then incubated with appropriate secondary antibodies for 1 h at 4 °C, then rinsed 3 times with PBS. DAB reagent (50–100 μl) was added and sections were observed under a microscope. The integrated optical density (IOD) of positively stained tissue was used to quantify the expression. The IOD of each tissue section was calculated from eight different 400× magnified fields.

### Masson’s trichrome staining

Samples were dewaxed and rehydrated using conventional methods, and then washed thoroughly with water. Sections were stained with haematoxylin from the Masson’s staining kit for 5 min, and then differentiated with ethanolic hydrochloric acid and rinsed thoroughly with water. Sections were then rinsed in running water until they turned a blue-black colour. The vermic acid red wine stain was added for 3–8 min, and then, sections were rinsed with distilled water and differentiated with 1% phosphomolybdic acid for 2 min. Finally, sections were stained with the aniline blue complex for 3 min, then differentiated with 1% glacial acetic acid for 1–2 min. Samples were sliced, dehydrated, rendered transparent with xylene and sealed with optical rubber. Sections were observed under a light microscope, and images were collected and used to calculate the volume fraction of collagen (CVF, CVF% = average collagen area/area of total field ×100).

### Western blotting

Atrial myocardium samples were weighed and treated with cold Radio-Immunoprecipitation Assay (RIPA) lysis buffer (1 ml/100 mg). Homogenized samples were centrifuged (12 000 rpm, 10 min, 4 °C) and a BCA protein assay kit (Yeason Biotech, Shanghai, China) was used to determine protein concentration. Loading buffer was added to the diluted supernatants to adjust the concentrations and volumes. Samples with equal protein content were then subjected to sodium dodecyl sulphate-polyacrylamide gel electrophoresis on a 10% polyacrylamide gel, and then transferred to a polyvinylidene difluoride membrane (Millipore, Boston, MA, USA). Membranes were blocked with blocking buffer containing 5% bovine serum albumin for 1 h at room temperature, followed by incubation with antibodies, anti-cTGF, anti-gal-3, anti-leptin, anti-vaspin (Abcam, Cambridge, UK), overnight at 4 °C. Anti-GAPDH was used as an internal control. Membranes were rinsed with TBST 3 times for 10 min each, followed by incubation with secondary antibodies for 1 h, and then washed three times with TBST for 15 min each. Finally, membranes were developed with chemiluminescence solution A and B mixed at a 1:1 ratio, along with the developing substrate. ImageJ software was used to calculate the relative optical density of the bands.

### Statistical analysis

Continuous data were expressed as mean ± standard deviation, while categorical data were expressed as percentages. The clinical characteristics of the two groups were compared using a Student’s t-test and chi-square test. Immunohistochemistry IOD and mRNA levels between groups were compared using Student’s t-test, while differences among the three types of adipose tissue were detected via one-way ANOVA with the Bonferroni test for post hoc examinations. The CVF% calculated from the Masson’s staining and the relative optical density of western blotting between the two groups were compared using Student’s t-test. A correlation analysis between mRNA expression and CVF% was conducted using univariate linear regression. A logistic regression model was used to determine risk factors for AF, while a linear regression model was used to explore the clinical parameters affecting the secretory profile of EAT. All data were analysed using SPSS 22.0 software (IBM, Almonte, NY, USA). Differences were considered significant when P < 0.05.

## Electronic supplementary material

Dataset 1
