# Supplementary material for: Human Epicardial Adipose Tissue cTGF Expression is an Independent Risk Factor for Atrial Fibrillation and Highly Associated with Atrial Fibrosis
Source: Sci Rep. 2018 Feb 26;8:3585. doi: 10.1038/s41598-018-21911-y (PMC5827202; doi:10.1038/s41598-018-21911-y)
Supplement: Supplementary file 1 — Dataset 1 [file 41598_2018_21911_MOESM1_ESM.doc]

Human Epicardial Adipose Tissue cTGF Expression is an Independent Risk Factor for Atrial Fibrillation and Highly Associated with Atrial Fibrosis

Qing Wang, PhD ^1¶^; Wang Xi, PhD ^1¶^; Liang Yin, MD ^1¶^; Jing Wang, MD ^1^; Hua Shen, PhD ^1^; Yang Gao, PhD ^1^; Jie Min, PhD ^1^; Yufeng Zhang, MD, PhD^1*^; Zhinong Wang, MD, PhD^1*^

1Center for Comprehensive Treatment of Atrial Fibrillation, Department of Cardiothoracic Surgery, Changzheng Hospital, Second Military Medical University, Shanghai, China

^*^corresponding Author: Yufeng Zhang, [zhyf19810824@163.com](mailto:zhyf19810824@163.com);

Zhinong Wang, wangzn007@163.com;

^¶^these authors contributed equally to this work

Supplementary Figure 1. Full-length gels and blots of western blotting results.


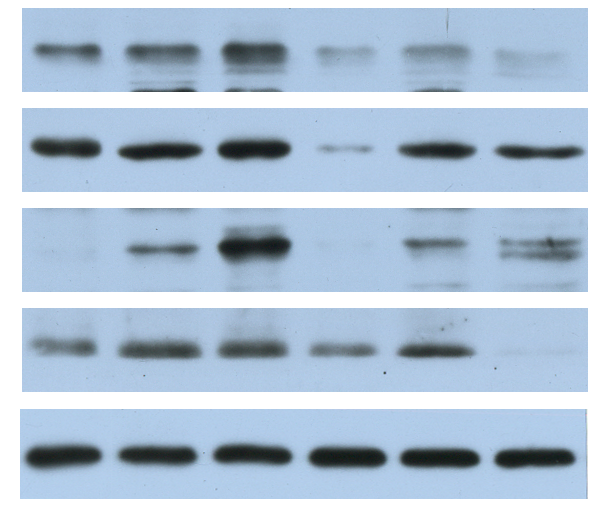


Supplementary Figure 2. Western blotting results of different exposure.


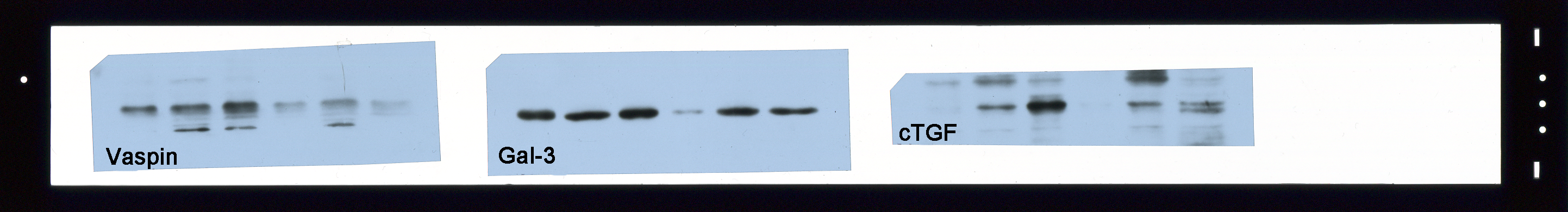

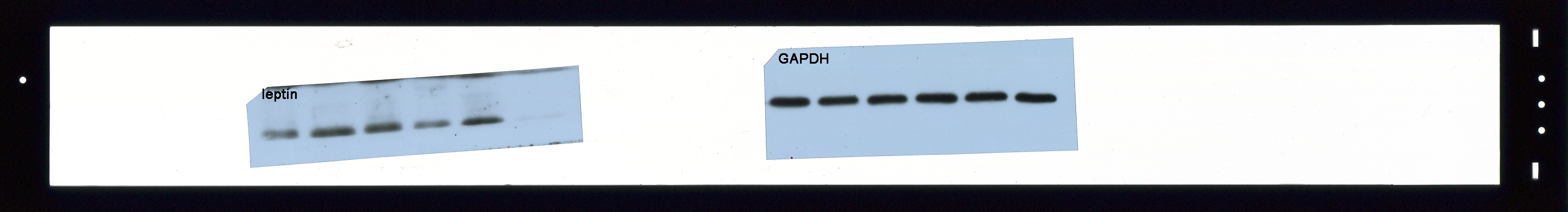


Supplementary Table S1 mRNA expression of cTGF, Gal-3. Leptin and Vaspin in adipose tissue of SR and AF patients

| Adipo-fibrokines | Adipose tissue type | SR group  (n=20) | AF group  (n=16) | t | *P* |
| --- | --- | --- | --- | --- | --- |
| cTGF | SAT | 0.57±0.25 | 0.49±0.32 | 0.804 | 0.427 |
|  | PAT | 2.88±1.61^**^ | 2.80±1.28 | 0.153 | 0.879 |
|  | EAT | 2.39±1.38^**^ | 10.23±5.69^**ΔΔ^ | 5.965 | <0.001 |
|  | F | 19.472 | 36.333 |  |  |
|  | *P* | <0.001 | <0.001 |  |  |
| Gal-3 | SAT | 1.15±0.55 | 1.00±0.45 | 0.928 | 0.360 |
|  | PAT | 2.19±1.83 | 2.39±1.44 | 0.359 | 0.722 |
|  | EAT | 4.26±2.18^**ΔΔ^ | 5.16±3.13^**ΔΔ^ | 1.021 | 0.314 |
|  | F | 17.741 | 17.831 |  |  |
|  | *P* | <0.001 | <0.001 |  |  |
| Leptin | SAT | 1.88±1.34 | 1.60±0.93 | 0.732 | 0.469 |
|  | PAT | 2.93±1.99 | 3.98±2.12^**^ | 1.522 | 0.137 |
|  | EAT | 0.98±0.52^ΔΔ^ | 2.43±1.34^Δ^ | 4.463 | <0.001 |
|  | F | 9.564 | 9.861 |  |  |
|  | *P* | <0.001 | <0.001 |  |  |
| Vaspin | SAT | 1.07±0.59 | 1.71±1.25 | 2.044 | 0.049 |
|  | PAT | 2.48±1.19^**^ | 3.28±1.99^*^ | 1.502 | 0.142 |
|  | EAT | 1.14±0.61^ΔΔ^ | 2.39±1.39 | 3.620 | 0.001 |
|  | F | 17.820 | 3.997 |  |  |
|  | *P* | <0.001 | 0.025 |  |  |

Post hoc examinations after one-way ANOVA. PAT vs SAT, EAT vs SAT, ^*^*P*<0.05, ^**^*P*<0.01; EAT vs PAT ^Δ^P<0.05, ^ΔΔ^P<0.01.

Supplementary Table S2 IOD of immunohistochemistry sections of adipose tissue of SR and AF patients

| Adipo-fibrokines | Adipose tissue type | SR group  (n=20) | AF group  (n=16) | t | *P* |
| --- | --- | --- | --- | --- | --- |
| cTGF | SAT | 5944.60±3464.64 | 6240.75±3001.37 | 0.270 | 0.789 |
|  | PAT | 22106.30±13862.08^**^ | 33461.50±13565.64^**^ | 0.846 | 0.019 |
|  | EAT | 27277.85±15141.24^**^ | 50487.00±30750.84^**^ | 2.963 | 0.006 |
|  | F | 17.144 | 20.977 |  |  |
|  | *P* | <0.001 | <0.001 |  |  |
| Gal-3 | SAT | 9429.50±6759.53 | 42892.19±22635.82 | 6.290 | <0.001 |
|  | PAT | 28648.00±18590.64^**^ | 52971.00±31834.76 | 2.866 | 0.007 |
|  | EAT | 41731.70±22332.22^**^ | 51277.87±30851.35 | 1.077 | 0.289 |
|  | F | 17.797 | 0.564 |  |  |
|  | *P* | <0.001 | 0.573 |  |  |
| Leptin | SAT | 20100.75±14691.53 | 15918.56±9779.85 | 0.980 | 0.334 |
|  | PAT | 30243.45±19967.75 | 39995.56±21544.66^**^ | 1.406 | 0.169 |
|  | EAT | 8232.70±5304.88^*ΔΔ^ | 28802.06±22296.77 | 4.000 | <0.001 |
|  | F | 11.331 | 6.592 |  |  |
|  | *P* | <0.001 | 0.003 |  |  |
| Vaspin | SAT | 10817.15±6346.68 | 16870.25±12899.51 | 1.843 | 0.074 |
|  | PAT | 23106.95±11089.41^**^ | 32425.06±19075.58 | 1.835 | 0.075 |
|  | EAT | 13638.10±15632.45^Δ^ | 41306.93±22977.96^**^ | 4.292 | <0.001 |
|  | F | 6.100 | 6.940 |  |  |
|  | *P* | 0.004 | 0.002 |  |  |

Post hoc examinations after one-way ANOVA. PAT vs SAT, EAT vs SAT, ^*^*P*<0.05, ^**^*P*<0.01; EAT vs PAT ^Δ^P<0.05, ^ΔΔ^P<0.01.

Supplementary Table S3 Relative optical density of western blotting bands of adipose tissue of SR and AF patients

| Adipo-fibrokines | Adipose tissue type | SR group  (n=20) | AF group  (n=16) | t | *P* |
| --- | --- | --- | --- | --- | --- |
| cTGF | SAT | 0.11±0.05 | 0.10±0.06 | 0.804 | 0.427 |
|  | PAT | 0.45±0.29^**^ | 0.66±0.26^**^ | 2.350 | 0.025 |
|  | EAT | 0.55±0.28^**^ | 0.98±0.63^**^ | 2.696 | 0.011 |
|  | F | 17.144 | 20.977 |  |  |
|  | *P* | <0.001 | <0.001 |  |  |
| Gal-3 | SAT | 0.20±0.14 | 0.87±0.45 | 6.389 | <0.001 |
|  | PAT | 0.57±0.36^**^ | 1.05±0.63 | 2.876 | 0.007 |
|  | EAT | 0.85±0.44^**Δ^ | 1.03±0.63 | 1.021 | 0.314 |
|  | F | 17.797 | 0.564 |  |  |
|  | *P* | <0.001 | 0.573 |  |  |
| Leptin | SAT | 0.38±0.27 | 0.32±0.19 | 0.732 | 0.469 |
|  | PAT | 0.59±0.40 | 0.80±0.42^**^ | 1.522 | 0.137 |
|  | EAT | 0.17±0.11^ΔΔ^ | 0.57±0.44 | 3.941 | <0.001 |
|  | F | 11.331 | 6.592 |  |  |
|  | *P* | <0.001 | 0.003 |  |  |
| Vaspin | SAT | 0.21±0.11 | 0.34±0.25 | 2.044 | 0.049 |
|  | PAT | 0.50±0.24^**^ | 0.66±0.40 | 1.502 | 0.142 |
|  | EAT | 0.29±0.30^Δ^ | 0.83±0.46^**^ | 4.202 | <0.001 |
|  | F | 6.100 | 6.940 |  |  |
|  | *P* | 0.004 | 0.002 |  |  |

Post hoc examinations after one-way ANOVA. PAT vs SAT, EAT vs SAT, ^*^*P*<0.05, ^**^*P*<0.01; EAT vs PAT ^Δ^P<0.05, ^ΔΔ^P<0.01.

Supplementary Table S4 Multilinear regression analysis of clinical parameters affecting the secretory profile of EAT

| Adipokines | Clinical parameters | B | SE | t | *P* |
| --- | --- | --- | --- | --- | --- |
| cTGF | AF | 8.247 | 1.681 | 4.907 | 0.000 |
|  | Gender | 0.245 | 1.587 | 0.155 | 0.879 |
|  | Age | -0.171 | 0.085 | -2.010 | 0.056 |
|  | Smoking | -0.102 | 1.663 | -0.061 | 0.952 |
|  | BMI | 0.163 | 0.371 | 0.438 | 0.665 |
|  | NYHA | 1.571 | 1.037 | 1.514 | 0.143 |
|  | LAD | -0.104 | 0.167 | -0.625 | 0.538 |
|  | T2DM | -0.946 | 1.673 | -0.565 | 0.577 |
|  | Hypertension | 0.078 | 1.912 | 0.041 | 0.968 |
|  | COPD | -0.741 | 2.164 | -0.342 | 0.735 |
|  | Stroke | -0.631 | 2.940 | -0.215 | 0.832 |
| Gal-3 | AF | 1.306 | 1.068 | 1.222 | 0.234 |
|  | Gender | -0.587 | 1.009 | -0.582 | 0.566 |
|  | Age | -0.038 | 0.054 | -0.703 | 0.489 |
|  | Smoking | 1.705 | 1.057 | 1.612 | 0.120 |
|  | BMI | 0.036 | 0.236 | 0.153 | 0.879 |
|  | NYHA | 1.325 | 0.660 | 2.009 | 0.056 |
|  | LAD | -0.087 | 0.106 | -0.822 | 0.419 |
|  | T2DM | 0.703 | 1.064 | 0.661 | 0.515 |
|  | Hypertension | -1.445 | 1.216 | -1.189 | 0.246 |
|  | COPD | -0.016 | 1.376 | -0.011 | 0.991 |
|  | Stroke | -1.089 | 1.869 | -0.582 | 0.566 |
| Leptin | AF | 1.864 | 0.410 | 4.544 | 0.000 |
|  | Gender | -0.175 | 0.387 | -0.451 | 0.656 |
|  | Age | 0.014 | 0.021 | 0.658 | 0.517 |
|  | Smoking | 0.438 | 0.406 | 1.079 | 0.291 |
|  | BMI | 0.077 | 0.091 | 0.854 | 0.401 |
|  | NYHA | -0.018 | 0.253 | -0.073 | 0.943 |
|  | LAD | -0.072 | 0.041 | -1.776 | 0.088 |
|  | T2DM | -0.098 | 0.408 | -0.240 | 0.813 |
|  | Hypertension | 0.415 | 0.467 | 0.888 | 0.383 |
|  | COPD | -0.286 | 0.528 | -0.541 | 0.593 |
|  | Stroke | -0.340 | 0.717 | -0.473 | 0.640 |
| Vaspin | AF | 1.082 | 0.428 | 2.526 | 0.019 |
|  | Gender | 0.491 | 0.404 | 1.215 | 0.236 |
|  | Age | 0.007 | 0.022 | 0.312 | 0.758 |
|  | Smoking | -0.713 | 0.424 | -1.683 | 0.105 |
|  | BMI | -0.043 | 0.094 | -0.455 | 0.653 |
|  | NYHA | -0.326 | 0.264 | -1.235 | 0.229 |
|  | LAD | 0.016 | 0.042 | 0.370 | 0.714 |
|  | T2DM | 0.523 | 0.426 | 1.228 | 0.231 |
|  | Hypertension | -0.502 | 0.487 | -1.031 | 0.313 |
|  | COPD | 0.647 | 0.551 | 1.174 | 0.252 |
|  | Stroke | 0.857 | 0.749 | 1.144 | 0.264 |
